# Predictors for length of hospital stay after inguinal hernia surgery

**Published:** 2015

**Authors:** S Aldoescu, T Patrascu, I Brezean

**Affiliations:** * General Surgery Clinic, “Dr. I. Cantacuzino” Hospital, Bucharest, Romania

**Keywords:** inguinal hernia, duration of hospital stay, complications, laparoscopic treatment

## Abstract

**Aim:** identifying the variables that can help in quantifying/ predicting duration of hospital stay after inguinal hernia surgery.

**Method:** 257 patients who were diagnosed with inguinal hernia underwent surgery between January 2013 and October 2014 and were prospectively registered and statistically analyzed by using linear regression with the aim of emphasizing, calculating and validating the predictors for duration of hospital stay.

**Results:** out of 257 patients, 50,7% underwent laparoscopic surgery (TAPP and TEP) and 49,7% had an anterior approach by using the technique described by Lichtenstein in most of the cases. From the variables registered in the study (age, recurrence, emergency surgery, ASA [American Society of Anesthesiologists] risk classification, surgery duration, local and general complications) only the age and presence/absence of complications were statistically associated with the modification of the duration of hospital stay in this pathology.

**Conclusions:** the duration of hospital stay can be evaluated preoperatory by using a mathematical model, which takes into consideration factors that depend on the patient or the procedure, with results that can have a significant impact on planning the local resources.

## Introduction

The surgical treatment of inguinal hernia represents one of the most frequent procedure in general surgery, reaching a rate of approximately 770.000 cases annually, registered only in the United States [**1**,**2**]. From 1959, many surgical treatment techniques have developed with the introduction of the alloplastic materials, culminating with the technique described by Lichtenstein in the 80’s, a technique that remained the standard treatment due to the decreased rates of recurrence. Introduction of laparoscopic techniques (at first TAPP and shortly after TEP) in the 90’ was received with great enthusiasm both by the persons directly involved, such as surgeons and patients, and by the systems of healthcare management. The advantages of the laparoscopic methods (the possibility of treating bilateral hernias by the same incisions, decrease of postoperative pain, decrease in the duration of hospital stay or return of patients to daily activities) have rapidly prevailed the costs of laparoscopic surgery, which were apparently high and mandatory implied general anesthesia or a very important learning curve [**3**-**6**]. Moreover, controversies have persisted both regarding the higher complications rates (the ones which were even inexistent in open surgery), as well as the higher number or recurrences as far as laparoscopic methods were concerned. However, data gathered in the last years have demonstrated that after completion of the learning curve, the results have been at least as good, this way the laparoscopic methods representing a suitable option both for the patient and for the hospital.

Although generally the surgical treatment of inguinal hernia needs short duration hospital stays, the post-operative complications but also the other factors that depend on the patient, can affect the post-operative course. The aim of this article is to identify significant variables with a role in prediction of hospital stay length for patients admitted with inguinal hernia.

## Material and Method

All patients over 18 years with diagnosis of primary or recurred unilateral or bilateral inguinal hernia were prospectively analyzed in General Surgery Clinic of “Dr. I. Cantacuzino” Hospital, between January 2013 and October 2014. Data were collected through individual questionnaires, separately from the observation chart, by an interviewing surgeon, different from the one who did the surgery. The variables registered for each patient included age, sex, associated comorbidities, type of hospital stay (programmed or emergency), ASA risk group, type of anesthesia, surgical procedure chosen and its duration, use/non-use of post-operative drainage, presence of post-operative complications, length of hospital stay and mortality rate.

The results were statistically analyzed by using SPSS 20 IBM (Chicago, Illinois) program. The linear regression was used to highlight and calculate the importance of predictors for the duration of the hospital stay. The “p” values of less than 0,05 were considered significant. 

## Results

257 patients were operated in the General Surgery Clinic of “Dr. I. Cantacuzino” Hospital between January 2013 and October 2014 by using both open surgery technique (anterior approach) and laparoscopic methods (TAPP and TEP). 

49,3% (127/ 257 patients) were operated on by an anterior approach: 117/ 127 by using the technique with alloplastic material described by Lichtenstein and 10/ 127 tissue surgical techniques. The rest of 130 patients out of 257 (50,6%) underwent laparoscopic surgery, by using TAPP technique in 123 cases and TEP technique in 7 cases. 

Demographic data and patients’ characteristics are shown in **Table 1**.

The average age of 127 patients from the group of open surgery technique was 63 years (SD=16). The average age of 130 patients who underwent laparoscopic surgery was significantly lower: 52 years (SD=16,6), p=0,0001. The difference between the ages was statistically significant and could be explained by the anesthesia type necessary in the laparoscopic approach, the old age most probably being a limiting factor for general anesthesia in safe conditions. 

The distribution of weight or height according to the two operative techniques was kept in close values, no statistically significant differences being registered. 

The distribution according to sex was the following: the percent of men was of 86,8% and of women of 13,2%. The most common type was indirect inguinal hernia, followed by the direct inguinal hernia.

The distribution according to the residence area, rural/ urban, did not register any statistically significant modifications for the two types of surgeries and kept the null hypothesis, p=0,232. 

**Table 1:** Demographic data and patients’ characteristics

Patients’ characteristics	Open surgery technique (n=127)		Laparoscopic surgery technique (n=130)	
	Medium ± SD		Medium ± SD	
Age	63 ± 16		52 ± 16,6	
Weight (kg)	74 ± 9,4		76 ±12,8	
Height (cm)	170 ± 7,7		172± 8,3	
Sex: M/ F	114/11		109/23	
	***N***		***N***	
Smoker	35		39	
COBP (Chronic Obstructive Bronchopneumopathy)	15		19	
Recurrence	15		17	
Type of effort:				
LOW/MEDIUM/HIGH	4/100/22		10/86/35	
Family history	30		42	
Job:				
RETIRED/EMPLOYEE/STUDENT/ UNEMPOLYED	80/40/2/4		45/68/2/15	
TYPE OF DEFECT	***N***	***%***	***N***	***%***
RECURRENCE	12	9.5	8	6.1
INDIRECT	62	49.2	70	53.4
FEMURAL	1	0.8	3	2.3
DIRECT	41	32.5	42	32.1
COMBINED	10	7.9	6	4.6
OTHERS	-	-	2	1.5
TOTAL	126	100.0	131	100.0
*Note:*				
a) *N = number of cases*				
b) SD = standard deviation. Standard deviation (SD) represents a measure of dispersion, in fact, the most common statistic parameter that measures data dispersion in case of a normal Gaussian type distribution and represents the absolute value of the difference as compared to the average one.				

The patients who underwent emergency surgeries (6 cases/ 257), although they were old (4/ 6 patients were aged over 80 years) did not register a significant post-operative complication rates, only 1 out of 6 cases being treated by laparoscopy using TAPP technique. However, age was correlated with post-operative complications rates, at the level p=0,05. Moreover, the distribution of post-operative complications is also significant for the two types of surgery techniques used: open and laparoscopic (**Table 2**). 

**Table 2:** The relationship between post-operative complications and surgery technique used (crosstabulation)

SURGERY TECHNIQUE – post-operative complications [Crosstabulation]					
			Post-operative complications		
			yes	no	Total
Surgery technique	Open surgery	Count	15	110	125
		%	**12,0%**	88,0%	100,0%
	Laparoscopic surgery	Count	4	128	132
		%	**3,0%**	97,0%	100,0%
Total		Count	19	238	257
		%	7,4%	92,6%	100,0%

Although it can be used together with the crosstabulation and the testing option of the chi-square hypothesis in the SPSS statistical analysis software, we preferred the U test of Mann-Whitney. We used the non-parametric test because it can be applied to categorical and nominal non-numerical data, being useful in areas in which the parametric tests are not operational. The result is established by consulting the p value (level of significance), which in SPSS is noted as “Sig.” or Significance. Accepted limits are 0,05 and 0,01. The decision rule is the following: if the value of “Sig.” in this column is less than the one of the chosen level of significance (this applies to our case, p=0,006), the null hypothesis will be rejected. 

Rejection of the null hypothesis is more important than its acceptance because this is a measurement capable of finding significant differences of probabilistic control specific to the significance interval of 95%. In the analysis below, we rejected the null hypothesis p=0,006. In conclusion, there is a statistically significant difference regarding the post-operative complications and the two surgery techniques used, the choice of laparoscopic surgery leading to a lower rate of complications (3% vs. 12%) from a statistical point of view.

In the case of predicting the length of the hospital stay after a laparoscopic surgery, the linear regression analysis was used and a statistic model through which factors with a significant influence on the length of the hospital stay, were identified. After verifying the independent factorial variables (post-operative complications, surgery technique and age), the aim of the regression was to identify those regression coefficients that would lead to lower prediction errors. Parameter B in **Table 3**, also called regression coefficient, shows the medium values “length of hospital stay” variable (positive or negative) takes when modifying with a unit the “post-operative complications” and “age” factorial variables.

**Table 3:** Medium values “the duration of hospital stay” variable takes when modifying with a unit the “post-operatory complications” and “age” factorial variables in laparoscopic surgery

Coefficients a, b								
Model		Unstandardized Coefficients		Standardized Coefficients	t	Sig.	95,0% Confidence Interval for B	
		B	Std. Error	Beta			Lower Bound	Upper Bound
1	**(Constant)**	**3,564**	1,551		2,943	,004	1,495	7,633
	Post-operative complications	**-1,496**	,728	-,161	-2,055	,042	-2,936	-,056
	Age	**,047**	,008	,436	5,554	,000	,030	,063
a. Dependant variable: DURATION OF HOSPITAL STAY (DAYS)								
b. Selected cases: SURGERY TECHNIQUE = LAPAROSCOPIC								

In conclusion, the equation which describes the relationship is y= b*x+a, where **a** is constant, as it can be seen in **Table 3**. 

For example, for a 72-year-old person, who underwent laparoscopic surgery and had postoperative complications, length of hospital stay was estimated to y = (0,047) * (72) + 3,564=6 days. Moreover, for a 40-year-old person, the length of hospital stay calculated according to the same model is estimated to 5 days. The coefficient B for post-operative complications is -1,49, which means that, on the average, if the laparoscopic technique is chosen, the length of hospital stay decreases with 1,4 days due to the lack of post-operative complications. 

**Fig. 1:**
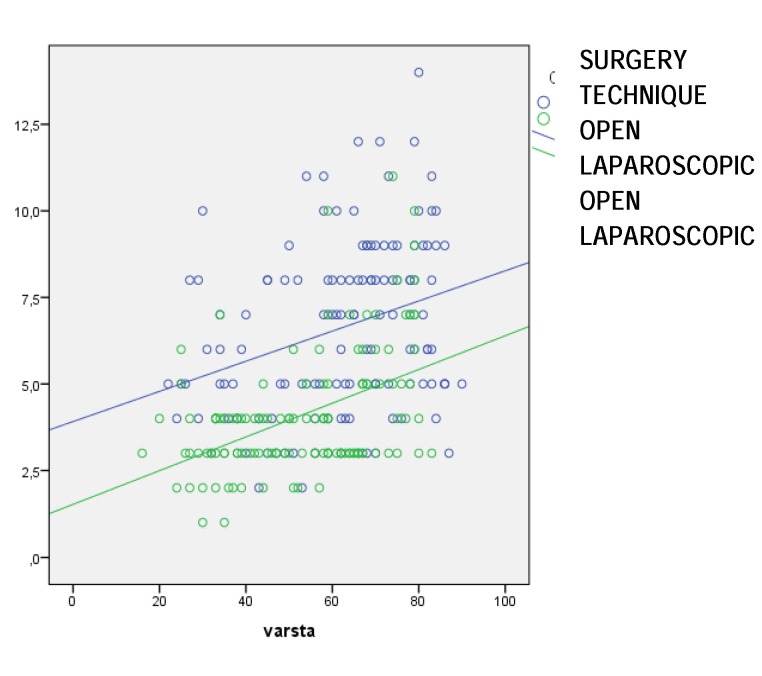
Length of hospital stay (days)

**Fig. 1** compares the two linear functions x = age, y = length of hospital stay with a selected variable: surgery technique (laparoscopic and open). The lines are oriented upwards which means that any rise of x = age, with a unit, is accompanied by a rise of y = duration of hospital stay, with a B constant (**Table 4**). The slope of the line is positive and the linear function is increasing. The scatterplot graphically highlights the selected variables in the model. 

The same linear regression model can predict the value it takes during the hospital stay also in the case of open surgery. If the length of hospital stay is y = 2*x, each time x (age) rises with one unit, the duration of the hospital stay rises with 2 units. 

For example, the length of the hospital stay estimated for a 72-year-old patient, who underwent open surgery with post-operative complications is y = (0,035)*(72)+6,785 = 9 days, and, for a 40-year-old patient, the length of hospital stay is estimated to be y = (0,035)*(40)+6,785 = 8 days. The lack of post-operative complications decreases duration of the hospital stay with 1,8 days in the described model.

The regression model set is valid and can be used for the analysis of the dependence between the variables. With a probability of 95% we can determine values/ prognoses of the length of hospital stay for a specific surgery technique, certain post-operative complications and the patients’ age [**7**-**11**]. 

## Discussions

The surgical treatment of inguinal hernia is currently the most frequent surgery performed by the general surgeon and, between the abdominal hernias, inguinal hernia is the most frequent [**12**,**13**].

Even if presently there are many surgical techniques approaching the inguinal wall defect, both anterior and posterior, no consensus has been reached regarding the ideal technique. If until the 90’s the technique described by Lichtenstein represented the treatment with the lowest rate of recurrences, the development of the minimally invasive techniques and the permanent assessment of experience have demonstrated these are accompanied by low rates of recurrence. In spite of the long-term favorable results, a study in 2009 suggested a rate of only 19,5% in some of the surgery centers in USA, the motivation being multifactorial but mainly based on financial costs and the laborious learning curve [**14**]. Although the best surgical treatment has not been determined yet, it is ideal that the surgeon knows different technical methods in order to answer to each patient individually. There are many studies in literature that have compared the laparoscopic approach with the open one, the results not being less controversial: if the study of Neumayer et al. reported the importance of open surgery as compared with the laparoscopic one, there are also many papers which proved the importance of the laparoscopic surgery as far as the quality of life and post-operative pain (aspects which are very important for patient) are concerned, but also the complications rates [**3**,**15**-**18**]. These aspects have also been confirmed by our study, the complications being found in 12% vs. 3% (open surgery vs. laparoscopic surgery). 

The same controversies exist regarding the costs of the hospital stay, the laparoscopic surgery being credited (by the advanced technique used) with higher costs for the hospital stay, but with a much faster socio-professional reintegration as compared with the open surgery (an approach which generates a cheaper hospital stay but with higher costs for the society through a higher convalescence period) [**19**,**20**]. However, beyond the different costs of the open surgery as compared with the laparoscopic one (being of 2840 lei vs. 3080 lei in our study), which the present study tried to demonstrate, was the existence of some confidence predictors which can help in the evaluation of costs but especially the length of hospital stay for the two surgical methods of treatment. The most accurate estimation for the length of the hospital stay may prove very useful in the efficient management of the occupancy of hospital beds but also the costs generated by this pathology. 

**Table 4:** Medium values “the duration of hospital stay” variable takes when modifying with a unit the “post-operatory complications” and “age” factorial variables in open surgery

Coefficients a, b								
Model		Unstandardized Coefficients		Standardized Coefficients	t	Sig.	95,0% Confidence Interval for B	
		B	Std. Error	Beta			Lower Bound	Upper Bound
1	**(Constant)**	**6,785**	1,373		5,669	,000	5,067	10,502
	Post-operatory complications	**-1,844**	,545	-,283	-3,384	,001	-2,923	-,766
	Age	**,035**	,012	,246	2,950	,004	,012	,059
a. Dependant variable: LENGTH OF HOSPITAL STAY (DAYS)								
b. Selected cases: SURGERY TECHNIQUE = OPEN								

The conclusion of this study is that although many variables seemed important in the first evaluation of the duration of hospital stay of a patient suffering from inguinal hernia, statistic analysis only validated “age” and the presence or absence of “post-operative complications” as predictors for the evaluation of the duration of hospital stay. 

Even if the prospective study is still in progress, at the moment the article will be published, the prospection for the recurrence evaluation and the long-term chronic post-operative painful syndrome will still be in progress. However, this does not bring modifications to the statistic results, an inconvenience of the statistic analysis being data collection from only one surgical center. For this, the reference to many surgical centers and to a higher number of surgeons who are experienced in this pathology would bring a plus value to the already existent data. 

Nevertheless, the current study may have significant clinical implications regarding planning of the occupancy of hospital beds in the department, planning of the number of procedures, estimation of the local costs per time unit at the level of the department but also at the level of the hospital. 
